# Targeting the autophagy-NAD axis protects against cell death in Niemann-Pick type C1 disease models

**DOI:** 10.1038/s41419-024-06770-y

**Published:** 2024-05-31

**Authors:** Tetsushi Kataura, Lucia Sedlackova, Congxin Sun, Gamze Kocak, Niall Wilson, Peter Banks, Faisal Hayat, Sergey Trushin, Eugenia Trushina, Oliver D. K. Maddocks, John E. Oblong, Satomi Miwa, Masaya Imoto, Shinji Saiki, Daniel Erskine, Marie E. Migaud, Sovan Sarkar, Viktor I. Korolchuk

**Affiliations:** 1 Biosciences Institute, Faculty of Medical Sciences, Newcastle University, Newcastle upon Tyne, NE4 5PL UK; 2 Department of Neurology, Institute of Medicine, University of Tsukuba, Tsukuba, Ibaraki 305-8575 Japan; 3 Institute of Cancer and Genomic Sciences, Institute of Biomedical Research, College of Medical and Dental Sciences, University of Birmingham, Birmingham, B15 2TT UK; 4 Mitchell Cancer Institute, Department of Pharmacology, F. P. Whiddon College of Medicine, University of South Alabama, Mobile, AL 36604 USA; 5 Department of Neurology, Mayo Clinic, 200 First St. SW, Rochester, MN 55905 USA; 6 Department of Molecular Pharmacology and Experimental Therapeutics, Mayo Clinic, 200 First St. SW, Rochester, MN 55905 USA; 7 Institute of Cancer Sciences, University of Glasgow, Glasgow, G61 1QH UK; 8 The Procter & Gamble Company, Cincinnati, OH 45040 USA; 9 Division for Development of Autophagy Modulating Drugs, Juntendo University Graduate School of Medicine, Bunkyo, Tokyo 113-8421 Japan; 10 Translational and Clinical Research Institute, Newcastle University, Newcastle upon Tyne, NE1 7RU UK; 11 Present Address: Centre for Genomic Regulation (CRG), The Barcelona Institute of Science and Technology, Barcelona, Spain

**Keywords:** Autophagy, Apoptosis, Neurodegenerative diseases, Metabolomics, Induced pluripotent stem cells

## Abstract

Impairment of autophagy leads to an accumulation of misfolded proteins and damaged organelles and has been implicated in plethora of human diseases. Loss of autophagy in actively respiring cells has also been shown to trigger metabolic collapse mediated by the depletion of nicotinamide adenine dinucleotide (NAD) pools, resulting in cell death. Here we found that the deficit in the autophagy-NAD axis underpins the loss of viability in cell models of a neurodegenerative lysosomal storage disorder, Niemann-Pick type C1 (NPC1) disease. Defective autophagic flux in NPC1 cells resulted in mitochondrial dysfunction due to impairment of mitophagy, leading to the depletion of both the reduced and oxidised forms of NAD as identified via metabolic profiling. Consequently, exhaustion of the NAD pools triggered mitochondrial depolarisation and apoptotic cell death. Our chemical screening identified two FDA-approved drugs, celecoxib and memantine, as autophagy activators which effectively restored autophagic flux, NAD levels, and cell viability of NPC1 cells. Of biomedical relevance, either pharmacological rescue of the autophagy deficiency or NAD precursor supplementation restored NAD levels and improved the viability of NPC1 patient fibroblasts and induced pluripotent stem cell (iPSC)-derived cortical neurons. Together, our findings identify the autophagy-NAD axis as a mechanism of cell death and a target for therapeutic interventions in NPC1 disease, with a potential relevance to other neurodegenerative disorders.

## Introduction

Autophagy is an intracellular degradation pathway mediated by sequestration of cellular components into autophagosomes followed by lysosomal degradation. This process is vital for cellular homeostasis by eliminating undesirable macromolecules like misfolded protein aggregates and damaged organelles such as mitochondria [1]. Genetic studies in mouse models demonstrated that brain-specific deletion of essential autophagy genes *Atg5* or *Atg7* display severe neurodegenerative and liver disease phenotypes [2–4]. In humans, mutations in *ATG7* result in detrimental neurodevelopmental disorders [5]. Likewise, compromised autophagy has been implicated in the pathology of myriad human disorders including a range of neurodegenerative and lysosomal storage diseases [6, 7]. Together, these findings suggest that autophagy is vital for cellular function and survival.

We recently identified the critical role of autophagy in the maintenance of NAD, a metabolite involved in a wide range of cellular bioenergetic, anabolic, and signalling pathways, and which was found to be the limiting factor in the survival of autophagy-deficient cells [8–10]. By employing an experimental knockout of the core autophagy machinery, we found that the loss of mitophagy, the key mitochondrial quality control mechanism, was the most upstream event in the detrimental cascade leading to cell death. Specifically, the impaired mitochondrial turnover resulted in an accumulation of dysfunctional mitochondria, increased generation of reactive oxygen species (ROS), elevated DNA damage levels, and the persistent activation of stress response pathways involving the NAD-consuming enzymes of the PARP and SIRT families [8–10]. The uncontrolled NAD consumption was found to be the primary mechanism for the depletion of the total NAD pools, leading to mitochondrial depolarisation via an insufficient supply of electrons from NADH, and consequently triggering an apoptotic phenotype [8, 11, 12]. Whilst this detrimental autophagy-NAD axis was evident in several models of autophagy deficiency from yeast to human neurons, it remains to be explored whether it plays a role in human pathologies.

NPC1 disease is a rare autosomal recessive lysosomal storage disorder with clinically heterogenous presentation, although typically characterised by progressive neurodegenerative dementia and hepatosplenomegaly [13]. NPC1 disease most frequently results from bi-allelic pathogenic variants in the *NPC1* gene encoding a polytopic transmembrane cholesterol transporter located on the lysosomal membrane. The mutant NPC1 protein is unstable and causes an accumulation of cholesterol and sphingolipids in the lysosomes [14–16]. We previously reported that loss of NPC1 function impairs autophagy by retarding autophagosome-lysosome fusion steps due to the failure in SNARE machinery [17]. Other studies also identified perturbation of autophagy in NPC1 cells due to a reduction in lysosomal degradative capacity, sphingosine kinase activity, and vascular endothelial growth factor (VEGF) levels [18, 19]. The impaired autophagic flux in NPC1 cells could be restored by the treatment with autophagy inducers such as rapamycin and Torin 1, inhibitors of the mammalian Target of Rapamycin (mTOR) [17, 19]. Notably, reactivation of autophagy by these drugs does not resolve the cholesterol accumulation in the lysosomes of NPC1 cells but is sufficient to rescue cell viability [17, 19, 20]. This suggests that the autophagy and cholesterol deficits in NPC1 cells are two pathogenic mechanisms acting in parallel, wherein the autophagy defect plays a key role in triggering cytotoxicity. Notably, we found that NAD supplementation could also improve the viability of NPC1 cells [8], which led us to investigate the role of the autophagy-NAD axis as an underlying mechanism and a potential therapeutic target in NPC1 disease.

Here, by performing metabolic profiling followed by mechanistic studies we identified the collapse of intracellular NAD pools as the main trigger of cell death in response to autophagy dysfunction in NPC1 cells. To test the therapeutic relevance of the autophagy-NAD axis, we identified FDA-approved drugs rescuing autophagy deficit in *Npc1*^*-/-*^ mouse embryonic fibroblasts (MEFs) via a chemical screen and also employed a range of bioavailable NAD precursor molecules. Both autophagy re-activation and supplementation with NAD precursors were sufficient to suppress cell death in *Npc1*^*-/-*^ MEFs, primary NPC1 patient fibroblasts, and neurons differentiated from NPC1 patient-derived iPSCs. These findings support the notion that dysfunctional autophagy, and specifically mitophagy, plays a causative role in the metabolic collapse of the actively respiring NPC1 cells. Together, our study provides a concept that targeting the autophagy-NAD axis can be a potential therapeutic strategy for NPC1 disease and other neurodegenerative conditions associated with autophagy and NAD defects.

## Results

### Metabolic deficits in respiring *Npc1*^*-/-*^ cells

**Fig. 1:**
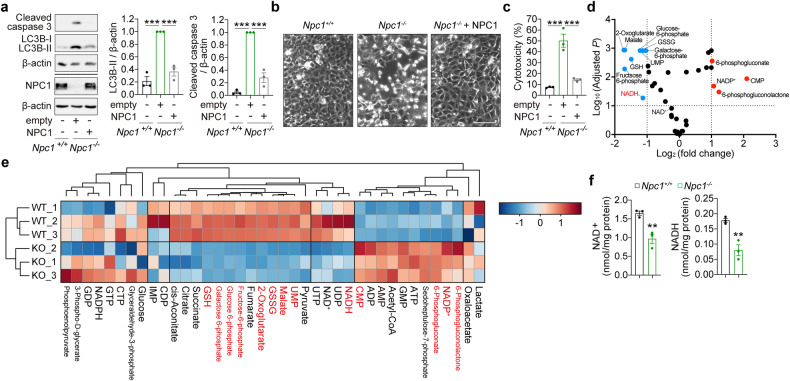
Metabolic deficits underlying apoptotic cell death in respiring *Npc1*^-/-^ cells. Immunoblot analyses for caspase-3 cleavage, LC3B lipidation and NPC1 expression (**a**), phase contrast images (**b**) and cytotoxicity assay results (**c**) of *Npc1*^*+/+*^ and *Npc1*^*-/-*^ MEFs transduced with empty or NPC1 WT, after 72 h (**a**, **b**) or 96 h (**c**) culture in galactose medium. **d, e**, Metabolomics analyses on *Npc1*^*+/+*^ and *Npc1*^*-/-*^ MEFs cultured in galactose medium for 48 h. **d**, Volcano plot representation of metabolites in a pairwise comparison of *Npc1*^*-/-*^ to *Npc1*^*+/+*^. Thresholds (|Log_2_(fold change)| > 1 and -log_10_(adjusted *P*) > 1) are shown as dashed lines. **e** Heatmap and clustering representations of analysed metabolites. Metabolites that change significantly **d** are highlighted in red. **f** Measurement of NAD^+^ and NADH levels in *Npc1*^*+/+*^ and *Npc1*^*-/-*^ MEFs after 60 h culture in galactose medium. Data are mean ± SEM of *n* = 3 biological replicates (**a, c, f**). *P* values were calculated by one-way ANOVA followed by multiple comparisons with the two-stage linear step-up procedure of Benjamini, Krieger and Yakutieli (**a, c**), multiple *t*-test with original FDR method of Benjamini and Hochberg (**d**) and unpaired two-tailed Student’s *t*-test (**f**). **P < 0.01; ***P < 0.001; with respect to *Npc1*^*+/+*^ MEFs or between the indicated groups. Scale bar: 200 µm (**b**).

*Npc1*^*-/-*^ MEFs from *Npc1*^*nih*^ mutant mice, which exhibit the clinical abnormalities of NPC1 disease, are viable in a standard cell culture medium [21]. Similar to the cells with a knockout of core autophagy genes (e.g., *Atg5*^*-/-*^), a metabolic shift from mitochondrial oxidative phosphorylation (OXPHOS) to glycolysis can be responsible for concealing the vulnerability of cells with the loss of *Npc1* [8, 22]. To stimulate mitochondrial respiration, we replaced glucose with galactose in a culture medium in order to force cells to use mitochondria for energy production [23]. Consistent with previous reports, *Npc1*^*-/-*^ but not wild-type (*Npc1*^*+/+*^) MEFs cultured in galactose medium for 72 h displayed an accumulation of LC3B-II (autophagosomes) due to block in autophagy flux (Fig. 1a) [17–20]. At the same time, we found significant activation of caspase-3 and cell death in *Npc1*^*-/-*^ MEFs (Fig. 1a–c). A similar cell death phenotype was observed in primary MEFs derived from a knock-in mouse carrying *Npc1*^*I1061T*^, the most common disease-associated mutation (Fig. S1a) [24]. Re-expression of wild-type NPC1 was sufficient to rescue the autophagy and cell death phenotypes in *Npc1*^*-/-*^ MEFs, thus validating the loss of NPC1 protein as the cause of autophagy deficit and subsequent cell death (Fig. 1a–c).

Our recent studies highlighted a metabolic deficit underlying cell death in *Atg5*^*-/-*^autophagy-deficient cells [8, 9]. To gain an insight into the mechanism of cell death in NPC1 disease, we analysed the levels of intracellular metabolites involved in bioenergetics pathways via metabolomics by comparing *Npc1*^*-/-*^ to *Npc1*^*+/+*^ MEFs before the onset of cell death (after 48 h culture in galactose media) (Figs. 1d, e, S1b). We identified alterations in glycolysis, tricarboxylic acid (TCA) cycle, pentose-phosphate pathway, and nucleotide metabolite levels in *Npc1*^*-/-*^ cells (Fig. 1d, e). Notably, NADH depletion, the key evolutionarily conserved metabolic deficit mediating cell death underlying autophagy deficiency [8, 9], was also found to be a feature of respiring *Npc1*^*-/-*^ MEFs (Fig. 1d, e). Targeted analysis of NAD^+^ and NADH levels using enzymatic assays confirmed that, similar to cells with the loss of core autophagy genes [8, 9], *Npc1*^*-/-*^ MEFs were characterised by depletion of the total NAD pool (Fig. 1f).

### Mitochondrial and mitophagy defects in respiring *Npc1*^*-/-*^ cells

**Fig. 2:**
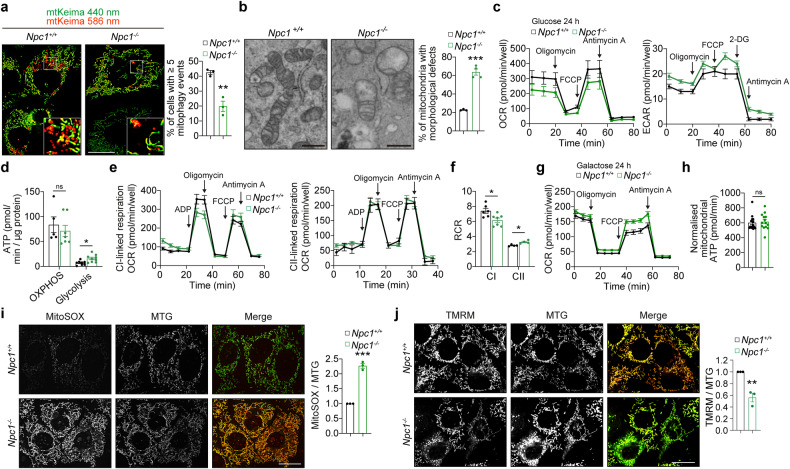
Mitochondrial deficits in *Npc1*^*-/-*^ cells. **a** Fluorescence microscopy images and quantification of mitophagy of *Npc1*^*+/+*^ and *Npc1*^*-/-*^ MEFs expressing mt-mKeima after 24 h culture in galactose medium. **b** transmission electron micrographs and the proportion of mitochondria with abnormal morphology of *Npc1*^*+/+*^ and *Npc1*^*-/-*^ MEFs cultured in galactose medium for 24 h. **c**–**h** Mitochondrial respiration and glycolytic function of *Npc1*^*+/+*^ and *Npc1*^*-/-*^ MEFs were analysed by measuring oxygen consumption rate (OCR) or extracellular acidification rates (ECAR), respectively. ECAR and/or OCR and ATP production rates in whole cell after 24 h culture either in glucose (**c**, **d**) or galactose medium (**g**, **h**). OCR in permeabilised cells treated with either CI or CII substrates (**e**, **f**) was measured. Respiratory control ratios (RCR) indicate a capacity for substrate oxidation and ATP turnover (**f**). **i** Live fluorescence microscopy images of *Npc1*^*+/+*^ and *Npc1*^*-/-*^ MEFs after 24 h culture in galactose medium, co-stained with MitoSOX and mitotracker green (MTG). **j** Confocal microscopy images of live *Npc1*^*+/+*^ and *Npc1*^*-/-*^ MEFs after 60 h culture in galactose medium, co-stained with TMRM and MTG. Data are mean ± SEM of n = 3-4 biological replicates as indicated (**a, b, i, j**) or n = 4-14 technical replicates (**c**–**h**) as indicated. *P* values were calculated by unpaired two-tailed Student’s *t*-test (**a, b, h**–**j**) and the multiple *t*-test with the two-stage linear step-up procedure of Benjamini, Krieger and Yakutieli (**d, f**). *P < 0.05; **P < 0.01; ***P < 0.001; ns (non-significant) with respect to *Npc1*^*+/+*^ MEFs. Scale bars: 20 µm (**a, i, j**); 500 nm (**b**).

We previously identified loss of mitochondrial quality control via mitophagy as the driver of mitochondrial and NAD deficits in *Atg5*^*-/-*^ MEFs [8]. Therefore, we investigated whether *Npc1*^*-/-*^ MEFs, which manifest with defective autophagy, recapitulate impairment of these processes. Mitophagy flux measured by mt-mKeima reporter at 586 nm excitation (indicating mitochondria in the acidic lysosomal environment [25]) was significantly reduced in *Npc1*^*-/-*^ MEFs compared to *Npc1*^*+/+*^ cells (Fig. 2a). Consistent with the impairment of the mitochondrial quality control, accumulation of mitochondria with abnormal morphology was also observed in *Npc1*^*-/-*^ MEFs (Fig. 2b). Furthermore, mitochondrial bioenergetics measured by Seahorse analyser indicated a shift towards glycolytic metabolism and increased mitochondrial Complex II activity at the expense of Complex I in *Npc1*^*-/-*^ MEFs (Fig. 2c–f). Interestingly, upon 24 h culture in galactose medium mitochondrial respiration in *Npc1*^*-/-*^ MEFs was restored to the level of *Npc1*^*+/+*^ cells (Fig. 2g, h). However, this activation of mitochondrial activity in *Npc1*^*-/-*^ MEFs was associated with the elevation of mitochondrial superoxide levels and, at the later stages of incubation in galactose medium (60 h), mitochondrial depolarisation (Fig. 2i, j). These data, together with the observation of a potentially compensatory hyperfusion of the mitochondrial network [26–28] in *Npc1*^*-/-*^ MEFs cultured for 24 h in glucose or galactose medium (Fig. 2a, i), provided further evidence of mitochondrial deficits in *Npc1*^*-/-*^ MEFs. Based on previous literature [8–10, 19], a plausible explanation for the observed phenotypes is that declining mitophagy flux in *Npc1*^*-/-*^ cells results in the accumulation of dysfunctional mitochondrial components (particularly Complex I which is vulnerable to oxidative stress), leading to the depletion of NAD pools, and loss of mitochondrial membrane potential.

### Identification of FDA-approved drugs rescuing defective autophagic flux in respiring *Npc1*^*-/-*^ cells

**Fig. 3:**
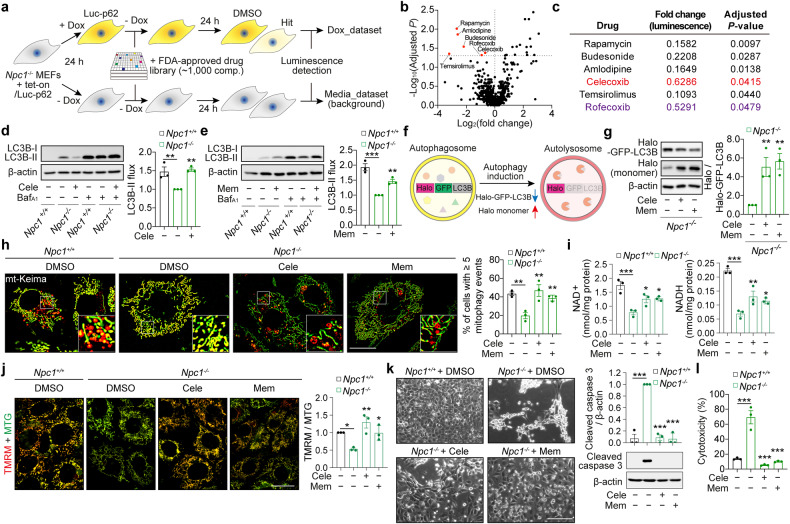
Autophagy inducers rescue NAD levels and cell death in *Npc1*^*-/-*^ cells. **a** Schematic illustration of chemical screen. **b, c** Volcano-plot representation of chemical screen results. Highlighted in red are hit compounds that reduce luminescence significantly (log2(FC) < 0 and –log_10_(adjusted *P*) > 1.3), and listed in (**c**). **d**, **e** Immunoblot analysis of autophagy flux in *Npc1*^*+/+*^ and *Npc1*^*-/-*^ MEFs cultured in galactose medium supplemented with 10 µM celecoxib (Cele) (**d**) or 30 µM memantine (Mem) (**e**) in the presence or absence of 100 nM bafilomycin A1 (Baf_A1_) for 24 h. **f** Schematic illustration of Halo processing assay. **g**, Halo processing assay in *Npc1*^*-/-*^ MEFs expressing Halo-GFP-LC3B cultured in galactose medium supplemented with Cele or Mem for 8 h. **h**, Fluorescence microscopy images and quantification of mitophagy of *Npc1*^*+/+*^ and *Npc1*^*-/-*^ MEFs expressing mt-mKeima 24 h after culture in galactose medium supplemented with Cele or Mem. Measurement of NAD^+^ and NADH levels (**i**), ΔΨm (**j**), Phase-contrast images and immunoblot analysis for caspase-3 cleavage (**k**), and cytotoxicity assay (**l**) in *Npc1*^*+/+*^ and *Npc1*^*-/-*^ MEFs cultured in galactose medium supplemented with Cele or Mem for 60 h (**i**, **j**), 72 h (**k**) or 96 h (**l**). Data are mean ± SEM of *n* = 3 biological replicates. *P* values were calculated by multiple *t*-test with FDR method of Benjamini and Hochberg (**b**, **c**) or by one-way ANOVA followed by multiple comparisons with the two-stage linear step-up procedure of Benjamini, Krieger, and Yakutieli (**d,** **e,** **g**-**l**). *P < 0.05; **P < 0.01; ***P < 0.001 with respect to untreated *Npc1*^*-/-*^ MEFs. Scale bars: 20 µm (**h, j**); 200 µm (**k**).

Unlike the situation with the loss of core autophagy genes (i.e., *Atg5*), autophagy deficit in *Npc1*^*-/-*^ cells can be rescued by autophagy activators such as rapamycin [17, 19, 20]. However, we found that rapamycin prevented cell death in both *Npc1*^*-/-*^ and *Atg5*^*-/-*^ MEFs, suggesting an autophagy-independent mechanism of cytoprotection by this pleiotropic drug via mTOR inhibition (Fig. S1c, d). To validate if pharmacological induction of autophagy is sufficient to rectify the defective autophagy-NAD axis in NPC1 disease, and to develop novel drug repurposing strategies, we screened a library of ~1,000 FDA-approved drugs to identify potent activators of autophagy in NPC1 cells. We employed *Npc1*^*-/-*^ MEFs with inducible expression of an autophagy substrate p62 (Luciferase-p62 reporter) to assess the ability of compounds to enhance p62 clearance (Fig. 3a) [29]. As a result, in addition to the mTOR inhibitors rapamycin and temsirolimus, two cyclooxygenase-2 (COX-2) inhibitors, celecoxib, and rofecoxib, were identified as the hits from the screen (Fig. 3b, c).

Since the neuroprotective effects of celecoxib have been reported, we decided to investigate celecoxib further [30, 31]. Additionally, we tested another FDA-approved drug memantine, which is clinically used for dementia and was previously identified by us as an autophagy activator [32, 33]. Immunoblotting analyses using the lysosomal inhibitor bafilomycin A_1_ (Baf_A1_) demonstrated that both drugs could rescue the impaired autophagy flux in *Npc1*^*-/-*^ MEFs (Fig. 3d, e) [34]. Autophagy-inducing activity of these compounds was validated by an alternative autophagy flux assay which measures the processing activity of Halo-GFP-LC3B reporter into Halo monomer via lysosomal degradation (Fig. 3f) [35]. Using this assay, both celecoxib and memantine were found to enhance the autophagy flux in *Npc1*^*-/-*^ MEFs (Fig. 3g). We did not observe any significant alterations in the phosphorylation of mTOR substrates, p70S6K (Thr389) and ULK1 (Ser757), suggesting that both drugs act as mTOR-independent autophagy inducers (Fig. S1e).

### Autophagy activators rescue mitophagy deficit, NAD depletion and cell death in *Npc1*^*-/-*^ MEFs

We next investigated whether restoring autophagic flux could rescue the cascade of downstream events within the autophagy-NAD axis [8, 9]. Treatment with celecoxib or memantine did not affect mitochondrial respiration but improved mitophagy activity in respiring *Npc1*^*-/-*^ MEFs, as evident by the increased portion of mitochondria in the acidic lysosomal environment compared to untreated *Npc1*^*-/-*^ cells (Figs. 3h, S1f, g). Concomitant with reactivation of mitophagy, celecoxib, and memantine restored NAD^+^ and NADH levels in respiring *Npc1*^*-/-*^ MEFs (Fig. 3i). Likewise, these drugs prevented mitochondrial depolarisation in NPC1 cells (Fig. 3j).

We further assessed the effects of autophagy inducers on cell viability. Both celecoxib and memantine suppressed caspase-3 activation and cell death in respiring *Npc1*^*-/-*^ MEFs (Fig. 3k, l). Unlike rapamycin, these drugs did not rescue cell death in *Atg5*^*-/-*^ MEFs, indicating autophagy-dependent rescue of cell viability in *Npc1*^*-/-*^ MEFs (Fig. S1h). Together, these data indicate that restoring autophagy and mitophagy capacity by celecoxib or memantine is beneficial for the maintenance of NAD levels, which promotes cell survival of *Npc1*^*-/-*^ cells.

### Boosting NAD levels rescues cell viability of *Npc1*^*-/-*^ MEFs

**Fig. 4:**
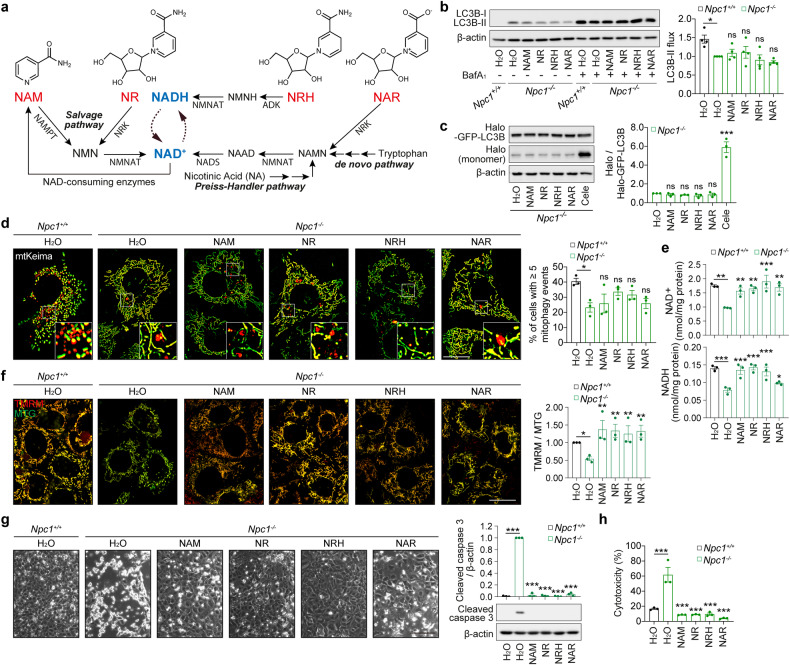
NAD precursor supplementation acts downstream of autophagy dysfunction, restores NAD levels and suppresses apoptotic cell death in *Npc1*^*-/-*^ cells. **a** Chemical structures of NAD precursors, nicotinamide (NAM), nicotinamide riboside (NR), dihydronicotinamide riboside (NRH), and nicotinic acid riboside (NAR) used in the study, and their conversion pathway into NAD(H) are shown. **b** Immunoblot analysis of autophagy flux in *Npc1*^*+/+*^ and *Npc1*^*-/-*^ MEFs cultured in galactose medium supplemented with 5 mM nicotinamide (NAM), 2 mM nicotinamide riboside (NR), 300 µM reduced nicotinamide riboside (NRH) and 50 µM nicotinic acid riboside (NAR) in the presence or absence of 100 nM bafilomycin A1 (Baf_A1_) for 24 h. **c** Halo processing assay in *Npc1*^*-/-*^ MEFs expressing Halo-GFP-LC3B cultured in galactose medium supplemented with the indicated NAD precursors for 8 h. **d** Fluorescence microscopy images and quantification of mitophagy of *Npc1*^*+/+*^ and *Npc1*^*-/-*^ MEFs cultured in galactose medium supplemented with the indicated NAD precursors for 24 h. Measurement of NAD^+^ and NADH levels (**e**), ΔΨm (**f**), phase-contrast images and immunoblot analysis for caspase-3 cleavage (**g**), and cytotoxicity assay (**h**) in *Npc1*^*+/+*^ and *Npc1*^*-/-*^ MEFs cultured in galactose medium supplemented with the indicated NAD precursors for 60 h (**e**, **f**), 72 h (**g**) or 96 h (**h**). Data are mean ± SEM of *n* = 3 (**c**–**h**) or 4 **b** biological replicates. *P* values were calculated by one-way ANOVA followed by multiple comparisons with the two-stage linear step-up procedure of Benjamini, Krieger, and Yakutieli. *P < 0.05; **P < 0.01; ***P < 0.001; ns (non-significant) with respect to untreated *Npc1*^*-/-*^ MEFs. Scale bars: 20 µm (**d, f**); 200 µm (**g**).

We previously showed that preventing NAD collapse in *Atg5*^*-/-*^ cells is sufficient to rescue the loss of mitochondrial membrane potential and cell death [8, 9]. Therefore, we investigated whether NAD depletion could be targeted to prevent the death of NPC1 cells. We tested four NAD precursors, nicotinamide (NAM), nicotinamide riboside (NR), dihydronicotinamide riboside (NRH), and nicotinic acid riboside (NAR), which are capable of replenishing the total NAD pool via different metabolic pathways (Fig. 4a) [36]. None of these NAD precursors were able to significantly restore defective autophagy or mitophagy flux in respiring *Npc1*^*-/-*^ MEFs, as assessed by LC3B blots in the absence or presence of bafilomycin A_1_, and with Halo-GFP-LC3B and mt-mKeima reporters, whilst celecoxib used as a positive control could rescue autophagy flux (Fig. 4b–d).

On the other hand, all the treatments efficiently replenished the diminishing levels of NAD^+^ and NADH in respiring *Npc1*^*-/-*^ MEFs (Fig. 4e), whilst mitochondrial respiration was not enhanced and even partially suppressed by the NAD precursors as previously reported (Fig S1i, j) [37, 38]. The rescue of NAD depletion was sufficient to prevent the loss of mitochondrial membrane potential (Fig. 4f). Accordingly, the NAD precursors suppressed caspase-3 activation and cell death in respiring *Npc1*^*-/-*^ MEFs (Fig. 4g, h). Taken together, we conclude that defective autophagy/mitophagy and the downstream metabolic NAD deficit are potential targets to prevent cell death caused by the loss of NPC1 function.

### Targeting the autophagy-NAD axis is cytoprotective in NPC1 patient-derived fibroblasts

**Fig. 5:**
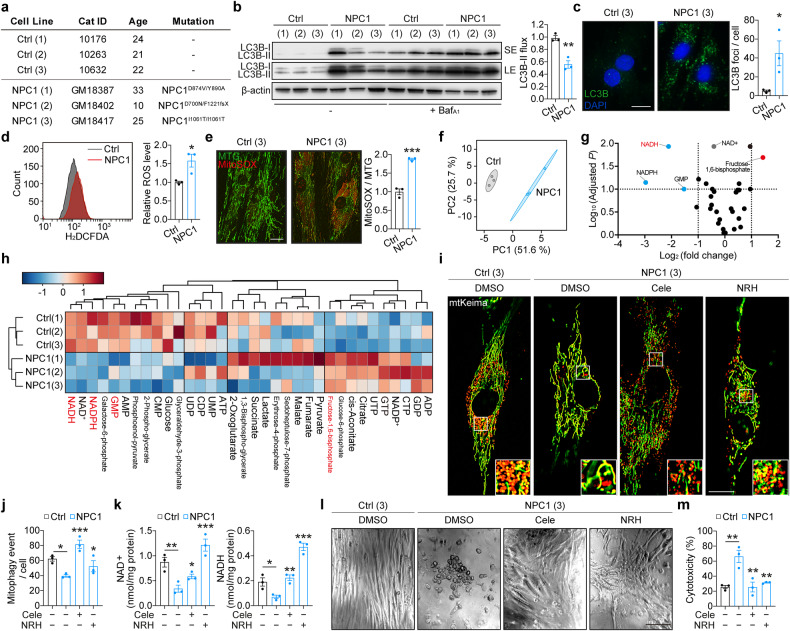
Restoring autophagy-NAD axis promotes cell survival of NPC1 patient-derived primary fibroblasts. **a** Overview of control and NPC1 patient-derived primary fibroblasts. **b** Immunoblot analyses of autophagy flux in control and NPC1 patient-derived primary fibroblasts cultured in galactose medium in the presence or absence of 100 nM Baf_A1_ for 24 h. **c** Representative confocal microscopy images and quantification of the number of puncta of immunofluorescence analysis with LC3B antibody in control and NPC1 patients-derived fibroblasts cultured in galactose medium for 7 days. **d** FACS analysis on cellular ROS levels by H_2_DCFDA staining in control and NPC1 patient-derived fibroblasts cultured in galactose medium for 7 days. **e** Fluorescence microscopy images of live primary fibroblasts in the same conditions as **d**, co-stained with MitoSOX and mitotracker green (MTG). **f**–**h** Metabolomics analyses on control and NPC1 patients-derived fibroblasts cultured in galactose medium for 7 days. **f** Principal component analysis (PCA) of metabolomics datasets. **g** Volcano plot representation of metabolites in a pairwise comparison of NPC1 to control fibroblasts. Thresholds (|Log_2_(fold change)| > 1 and -log_10_(adjusted *P*) > 1) are shown as dashed lines. **h** Heatmap and clustering representations of analysed metabolites. Metabolites that change significantly (**e**) are highlighted in red. Fluorescence microscopy images (**i**) and quantification of mitophagy (**j**) in control and patient-derived primary fibroblasts cultured in galactose medium supplemented with 10 µM Cele or 100 µM NRH for 24 h. **k** Measurement of NAD^+^ and NADH levels in control and patient-derived primary fibroblasts cultured in galactose medium supplemented with Cele or NRH for 7 days. **l**, **m** Cytotoxicity assay (**k**) and phase-contrast images (**l**) in control and patient-derived primary fibroblasts cultured in galactose medium supplemented with Cele or NRH for 7 days and challenged with 500 µM H_2_O_2_ in serum free medium for 2 h. Data are mean ± SEM of *n* = 3 cell lines per group (**b**–**e, j**, **k**, **m**). *P* values were calculated by unpaired two-tailed Student’s *t*-test (**b–e**), multiple *t*-test with the original FDR method of Benjamini and Hochberg (**g**), or by one-way ANOVA followed by multiple comparisons with the two-stage linear step-up procedure of Benjamini, Krieger and Yakutieli (**j**, **k**, **m**). *P < 0.05; **P < 0.01; ***P < 0.001 with respect to control (**b–e**) or untreated NPC1 primary fibroblasts (**j**, **k**, **m**). Scale bars: 20 µm (**c, e, i**); 200 µm (**l**).

To test the relevance of the dysfunctional autophagy-NAD axis in NPC1 patient-derived cellular models, we initially employed primary patient fibroblasts carrying different NPC1 mutations as well as control lines (Fig. 5a). Similar to the experiments with MEFs described above, patient-derived fibroblasts were cultured in galactose media to enforce mitochondrial respiration and expose any mitochondrial deficits. Consistent with previous reports, NPC1 patient fibroblasts were characterised by a dampened autophagy flux with an accumulation of autophagosomes as well as elevated oxidative stress measured by general ROS indicator CM-H_2_DCFDA and MitoSOX staining (Fig. 5b–e) [17, 39]. In agreement with the data in *Npc1*^*-/-*^ MEFs (Fig. 1d, e, S1b), metabolomics analyses of control and NPC1 patient fibroblasts identified NADH deficit as well as perturbation in glycolytic metabolites and nucleotides (Fig. 5f–h). Furthermore, consistent with the cellular phonotypes of *Npc1*^*-/-*^ MEFs, mitophagy flux and NAD levels were lower in NPC1 patient fibroblasts (Fig. 5i–k).

We next tested pharmacological interventions targeting the autophagy-NAD axis in NPC1 patient fibroblasts. We selected celecoxib and NRH as the two most potent interventions to restore autophagy and NAD levels, respectively, as per our data. Celecoxib, and to a lesser extent NRH, rescued the mitophagy decline due to the loss of NPC1 function in respiring patient fibroblasts (Fig. 5i, j). This discrepancy in the effect of NRH on mitophagy flux could be due to cell type differences where primary cells but not MEFs express detectable levels of PINK1, which is required for the activation of mitophagy by NAD precursors [40–42]. At the same time, both celecoxib and NRH increased the NAD levels in NPC1 patient fibroblasts (Fig. 5k). Unlike *Npc1*^*-/-*^ MEFs, culturing patient-derived fibroblasts in galactose medium did not result in a strong cell death phenotype. Since autophagy can serve as a defence mechanism against oxidative stress which has been demonstrated to occur in NPC1 disease [43–46], we introduced further oxidative stress by the treatment with low levels of H_2_O_2_. In these conditions, which were tolerated by the control cells, NPC1 patient fibroblasts exhibited substantial cell death that was effectively rescued by treatment with celecoxib or NRH (Fig. 5l, m).

### Targeting the autophagy-NAD axis improves the survival of NPC1 patient iPSC-derived neurons

**Fig. 6:**
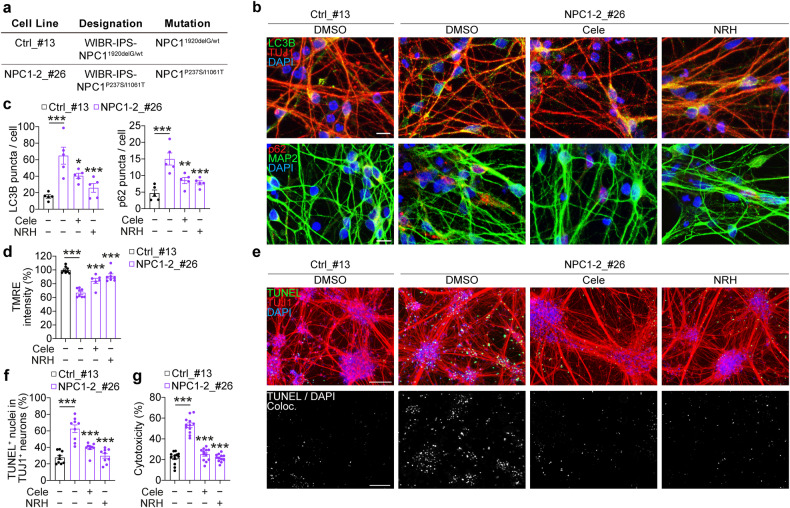
Restoring autophagy-NAD axis protects against cell death of NPC1 patient iPSC-derived cortical neurons. **a** Overview of control and NPC1 patient-derived iPSCs. Immunofluorescence images (**b**) and quantification of LC3B and p62 puncta in TUJ1^+^ or MAP2^+^ cells (**c**) in control (Ctrl_#13) and NPC1 patient (NPC1-2_#26) iPSC-derived cortical neurons after 4 weeks of neuronal differentiation, where NPC1 neurons were treated with 10 µM Cele or 100 µM NRH for the last 6 days. **d** TMRE fluorescence intensity (percentage of pre- and post-FCCP treatment) for ΔΨm in Ctrl_#13 and NPC1-2_#26 iPSC-derived cortical neurons in the same conditions as (**b**, **c**). Fluorescence images (**e**) and quantification of TUNEL^+^ apoptotic nuclei in TUJ1^+^ cells (**f**), and cytotoxicity assay results (**g**) in Ctrl_#13 and NPC1-2_#26 iPSC-derived cortical neurons in the same conditions as (**a**, **b**). Data are mean ± SEM of *n* = 5 (**c**), 6 (**d**, treated conditions), 8 (**d**, untreated conditions), 9 (**f**) or 12 (**g**) biological replicates. *P* values were calculated by one-way ANOVA followed by multiple comparisons with the two-stage linear step-up procedure of Benjamini, Krieger, and Yakutieli. *P < 0.05; **P < 0.01; ***P < 0.001 with respect to untreated *NPC1* mutant cells. Scale bars: 10 µm (**b**); 100 µm (**e**).

To evaluate the biomedical importance of the dysfunctional autophagy-NAD axis in clinically relevant cellular platforms, we employed iPSCs derived from a NPC1 disease patient and a healthy control that were differentiated into cortical neurons via established culture conditions (Fig. 6a) [20, 47]. The iPSC lines have been previously characterised [20], and the cellular identity of the control and NPC1 patient iPSC-derived neurons was confirmed by expression of neuronal markers, TUJ1 and MAP2 (Fig. 6b). As reported by us previously, patient-derived neurons carrying the NPC1 mutation were characterised by an autophagy deficit visualised by the accumulation of LC3B (autophagosomes) and p62 (autophagy substrate) puncta in TUJ1 or MAP2 positive neuronal cells (Fig. 6b, c) [8, 34]. We have also shown that NPC1 iPSC-derived neurons exhibited lower cell viability and NAD levels compared to the control neurons [8, 20]. Additionally, we found mitochondrial depolarisation in NPC1 neurons, and further confirmed the cell death phenotype by cytotoxicity assay and TUNEL-positive apoptotic nuclei in TUJ1-positive neurons after 4 weeks of neuronal differentiation in standard culture conditions (Fig. 6d–g). These data highlight the defective autophagy-NAD axis that correlates with increased cell death at basal state in NPC1 patient iPSC-derived neurons.

**Fig. 7:**
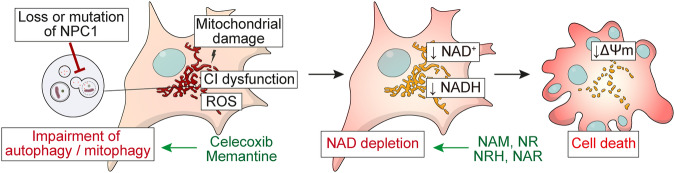
Schematic representation of the present study. Loss of function in NPC1 results in autophagy/mitophagy dysfunction leading to NAD depletion and subsequent mitochondrial depolarisation and apoptotic cell death. Autophagy inducers and NAD precursors (green) can ameliorate the phenotypes in mouse and human NPC1 model cells.

Therefore, we targeted the autophagy-NAD axis with pharmacological activators of the pathway to test their therapeutic efficacy in iPSC-derived, disease-relevant neuronal cells. Treatments with celecoxib and NRH suppressed an accumulation of autophagy markers LC3B and p62 puncta, indicating restoration of autophagy flux in NPC1 patient iPSC-derived neurons (Fig. 6b, c). Furthermore, both celecoxib and NRH rescued the mitochondrial depolarisation and cell death phenotypes in NPC1 patient-derived neurons (Fig. 6d–g). Together, we conclude that targeting the autophagy-NAD axis, either by interventions rescuing autophagy deficit or by preventing NAD depletion, could be a viable therapeutic strategy in NPC1 disease (Fig. 7).

## Discussion

In addition to the previous findings that loss of NPC1 function results in impaired autophagy, mitochondrial dysfunction, and increased cell death [17–20, 39, 48, 49], we identified a specific metabolic signature, namely NAD depletion, as a feature of NPC1 cell models. These defects in the autophagy-NAD axis could trigger mitochondrial depolarisation and subsequent apoptotic cell death, as previously shown in autophagy-deficient (*ATG5*^*-/-*^) genetic models [8, 9]. Importantly, unlike *ATG5*^*-/-*^ cells, autophagy function in NPC1 cells can be restored by pharmacological autophagy activators [17, 20].

To test if restoring autophagy improves the NAD deficit, we initially used the mTOR inhibitor rapamycin. However, rapamycin prevented cell death not only in *Npc1*^*-/-*^ MEFs but also in *Atg5*^*-/-*^ MEFs, implying an autophagy-independent mechanism. This effect of rapamycin could be due to amelioration of translational burden in the cells through mTORC1 inhibition [19]. In contrast, celecoxib and memantine, which we identified in this and previous studies as autophagy inducers by screening FDA-approved drugs, exhibited mTOR-independent but strictly autophagy-dependent cytoprotective activities [32, 33]. Whilst memantine has been indicated to modulate autophagy via activation of unfolded protein response pathway [33], the mechanism by which celecoxib induces autophagy is not known. Since our screen also identified another COX-2 inhibitor rofecoxib as a hit, these compounds may activate autophagy by COX-2 inhibition through an unknown mechanism. However, we cannot exclude the possibility of a COX-2-independent mechanism [50].

NAD precursor supplementation suppressed mitochondrial respiration in *Npc1*^*-/-*^ MEFs which was previously suggested to be due to improved efficiency of the electron transfer chain [37, 38]. NAD boosting also protected *Npc1*^*-/-*^ MEFs from cell death without restoring the autophagy/mitophagy deficit, indicating that the decline in NAD levels is downstream of dampened autophagy in these cells. On the other hand, mitophagy in NPC1 patient-derived fibroblasts was partially rescued by boosting NAD levels. Given that NAD precursors have been suggested to require PINK1 to activate mitophagy, this might be explained by the high expression levels of PINK1 and Parkin in primary fibroblasts whilst these are undetectable in cultured MEFs [40–42]. Similarly, autophagy function in NPC1 patient iPSC-derived cortical neurons was restored by NAD replenishment. Recent studies demonstrated a link between mitochondrial homeostasis and proteostasis, which can be improved by NAD boosters in neurons [9, 51, 52]. Indeed, our previous study identified that autophagy-deficient *ATG5*^*-/-*^ neurons display the accumulation of aggregated proteins, indicating proteostasis dysregulation [9]. NAD supplementation could rescue this impaired proteostasis, which is concomitant with mitochondrial function [9]. We proposed that this could be due to changes in ATP levels as we identified significant ATP depletion in the neurons and V-ATPase proton pump requires ATP for the maintenance of lysosomal acidity which is essential for optimal enzyme function [9, 53]. It should be noted that in contrast to *ATG5*^*-/-*^ cells, autophagy in NPC1-mutant neurons is not fully dysfunctional and can be reactivated. Together, NAD precursor supplementation restores NAD levels leading to the improvement of mitochondrial function and subsequent ATP generation, which may replenish lysosomal degradation capacity and thereby result in restoration of autophagic function in the NPC1-mutant neurons. On the other hand, ATP levels in *Npc1*^-/-^ MEFs and NPC1 patient-derived primary fibroblasts were not significantly altered, which may underlie the fact that NAD supplementation exhibited no or mild upregulation of autophagy function in MEFs and primary fibroblasts, respectively. However, we cannot rule out any other possibility for this discrepancy, such as that NAD^+^-dependent enzymes including SIRT1 known to regulate autophagy have a greater role in the maintenance of autophagy flux in neurons [10]. Together, future investigations on autophagy regulation by NAD metabolism in different cell types across species are required to fully delineate the crosstalk between autophagy and NAD in the regulation of cellular homeostasis.

Loss of NPC1 function results in the accumulation of lipids including cholesterol and sphingolipids in the late endosomal/lysosomal compartments [54]. It has been suggested that abnormal cholesterol storage leads to dysfunction in lysosomes and other cellular compartments such as endoplasmic reticulum, Golgi, and mitochondria, and contributes to neurological pathologies in NPC1 disease [54, 55]. We and others previously reported that stimulating autophagy was unable to rescue the cholesterol phenotypes in NPC1 cells whilst a low dose of cholesterol-lowering agent HP-β-cyclodextrin induced the partial depletion of cholesterol without perturbing autophagy [17, 19]. Therefore, combinations of autophagy activators, NAD boosters, and cholesterol-lowering agents may synergistically improve cellular homeostasis in NPC1 disease.

In summary, we found that restoring autophagy activity or boosting NAD levels efficiently prevents cell death in models of NPC1 disease. We also demonstrated the efficacy of celecoxib in the preclinical cellular models using NPC1 patient iPSC-derived neurons, highlighting this drug as a candidate for repositioning. Furthermore, NRH, a potent and bioavailable NAD precursor, also strongly restored cellular dysfunction in NPC1 cells. Together our findings provide a concept that autophagy-NAD axis could be exploited as a therapeutic target in Niemann-Pick type C1 disease. Given the importance of autophagy for neuronal survival, this approach may also be generalizable to other neurodegenerative conditions associated with autophagy and NAD defects.

## Materials and Methods

### Culture of mammalian cell models

Immortalized *Npc1*^+/+^ and *Npc1*^*-/-*^ MEFs (gift from Peter Lobel) [21], p62-luciferase expressing *Npc1*^*-/-*^ MEFs [29], *Atg5*^*+/+*^ and *Atg5*^*-/-*^ MEFs (gift from Noboru Mizushima) [56] and primary *Npc1*^*WT*^ and *Npc1*^*I1061T*^ (gift from Daniel Ory) [24] MEFs were maintained in DMEM supplemented with 10% foetal bovine serum (FBS), 100 U/mL penicillin/streptomycin and 2 mM L-glutamine (all from Sigma-Aldrich) at 37 °C, and 5% CO_2_ in a humidified incubator. 293FT cells (Invitrogen, R70007) were cultured as above in a medium supplemented with 1X MEM non-essential amino acids (Gibco). 293GPG cells (gift from Daniel Ory) were cultured as above in a medium supplemented with 1X MEM non-essential amino acid solution, 1 μg/mL tetracycline (Sigma-Aldrich), 2 µg/mL puromycin (Gibco), and 0.3 mg/mL G418 (Gibco) (referred to as 293GPG medium). Human primary fibroblasts derived from control (10263, 10632, 10763, gift from Devin Oglesbee) and NPC1 patient (GM18387, GM18402, GM18417, obtained from Coriell Cell Repositories) were maintained as above in medium supplemented with 15% FBS. Cell authentication was not performed. Mycoplasma contamination was routinely tested using MycoAlert assay kit (Lonza).

### iPSC culture and neuronal differentiation

Human induced pluripotent stem cell (iPSC) lines, control_#13 (WIBR-IPS-NPC11920delG/wt, clone #13) and NPC1-2_#26 (WIBR-IPS-NPC1P237S/I1061T, clone #26) iPSC lines were cultured as previously described [20, 57]. The human iPSC lines were cultured on inactivated mouse embryonic fibroblast (MEF) feeder layer in hESC medium comprised of DMEM/F-12, 5% KnockOut Serum Replacement, 1% L-glutamine, 1% non-essential amino acids, 1% penicillin/streptomycin, 4 ng/mL human recombinant basic fibroblast growth factor (bFGF) (all from Gibco), 15% FBS (HyClone) and 0.1 mM β-mercaptoethanol (Sigma-Aldrich) and maintained in a humidified incubator with 5% CO2 and 5% O2 at 37 °C. For experimentation, the iPSCs were cultured feeder-free on Geltrex basement membrane matrix in StemFlex Basal Medium supplemented with StemFlex 10X Supplement (all from Gibco).

Neural stem cells (NSCs) were differentiated from iPSCs, as described previously [58, 59]. NSCs were cultured on Poly-L-ornithine and Laminin (PO-L) (Sigma-Aldrich) coated plates or flasks in N2B27 medium comprised of DMEM/F-12 and Neurobasal medium in 1:1 ratio, 1% N-2 supplement, 2% B-27 supplement, 1% penicillin/streptomycin (all from Gibco), 0.1% β-mercaptoethanol (Sigma-Aldrich) supplemented with 10 ng/mL FGF-2 (Miltenyi Biotec) and 10 ng/mL EGF (PeproTech), and were maintained in a humidified incubator with 5% CO_2_ at 37 °C. NSCs were passaged twice a week with 0.05% Trypsin-EDTA (Gibco), and the medium was changed on alternate days.

Neuronal differentiation of human iPSC-derived NSCs was carried out as described previously [58, 59]. The NSCs were seeded as above on PO-L coated plates in N2B27 medium without FGF-2 and EGF. At day 4 of neuronal differentiation, cells were treated with 10 µM DAPT (Tocris) to prevent cell proliferation. The N2B27 medium (without FGF-2 and EGF) was changed every 2 days and neuronal differentiation was carried out for 4 weeks. The neurons generated in vitro were cortical in nature [58, 59].

The control and NPC1 patient-derived iPSCs were originally generated in the lab of Rudolf Jaenisch at the Whitehead Institute for Biomedical Research. These cell lines were used for this study in the lab of Sovan Sarkar at the University of Birmingham under material transfer agreements, UBMTA 15-0593 and UBMTA 15-0594. All experiments were performed in accordance with ISSCR and institutional guidelines and regulations.

### Generation of stable cell lines

Re-introduction of the NPC1 gene into *Npc1*^*-/-*^ MEFs was achieved by retroviral transduction. For retrovirus production, 293GPG cells were seeded in a 10 cm dish (5.5 × 10^6^ cells/10 mL/dish) 48 h prior to DNA transfection. At 90% confluency, the culture medium was replaced with tetracycline-free 293GPG medium and transfected with either empty or NPC1 retroviral expression plasmid (gift from Daniel Ory) with 4 μg of DNA using Lipofectamine 2000 (Thermo Fisher Scientific) according to manufacturer instruction. Following overnight incubation, the medium was replaced with 293GPG medium containing 20% FBS. Virus-containing medium was collected 48 h post-transfection and was filtered through a 0.45 μm membrane filter and overlaid on 70% confluent cells in the presence of 10 μg/mL polybrene (Sigma-Aldrich).

Generation of cells stably expressing YFP-Parkin, mt-mKeima, or Halo-GFP-LC3B was achieved by packaging retroviruses in 293FT cells. Cells were seeded in a 10 cm dish (6.0 × 10^6^ cells/10 mL/dish) in antibiotic-free culture medium. Next day, cells were transfected with plasmids containing the packaging gag/pol and envelope pCMV-VSV-G genes, and the YFP-Parkin-IRES-zeo (gift from Douglas Green and Stephen Tait, Addgene, 61728) [60], pCHAC-mt-mKeima (gift from Richard Youle, Addgene, 72342) [61] or pMRX-No-HaloTag7-mGFP-LC3B (gift from Noboru Mizushima, Addgene, 184901) [35] using Lipofectamine 2000. Following overnight transfection, the medium was replaced with a fresh antibiotic-free medium that was collected after 24 h. Virus-containing medium was collected and used for transduction as above.

### Galactose medium culture and supplementation

To induce mitochondrial respiration in MEFs and human primary fibroblasts, cells were cultured in galactose medium (glucose-free DMEM (Gibco) supplemented with 10 mM D-galactose, 10 mM HEPES, 1 mM sodium pyruvate, 4 mM L-glutamine, 100 U/mL penicillin/streptomycin and 10% FBS (all from Sigma-Aldrich) 24 h post seeding. Galactose medium was supplemented with various compounds as indicated in figure legends: 100 nM bafilomycin A_1_ (Baf_A1_, Enzo Life Sciences, BML-CM110-0100), 10 µM celecoxib (Cele, Sigma-Aldrich, SML3031), 30 µM memantine (Mem, Sigma-Aldrich, M9292), 5 mM nicotinamide (NAM, Sigma-Aldrich, N0636), 2 mM nicotinamide riboside (NR, ChromaDex), 100 or 300 µM reduced nicotinamide riboside (NRH) [36] and 50 µM nicotinic acid riboside (NAR) [36]. All compound supplements were added at 0 h.

### Screening of autophagy inducers

p62-luciferase expressing *Npc1*^*-/-*^ MEFs were plated into white walled 384 well plates (0.2 × 10^4^ cells/20 µL/well). Cells were then cultured in DMEM supplemented with or without 1 µg/mL doxycycline (Sigma-Aldrich) for 24 h. After three washes with PBS, cells were treated with 935 compounds (10 µM) from an FDA-approved drug library (LifeArc) for 24 h. Following the treatment, 5 µL of Bright-Glo (Promega) was added to each well, and luminescence was assessed on a BMG Omega POLARstar plate reader. Luminescence fold change (treated to control) was calculated using adjusted luminescence signals by subtracting background signals (-Dox datasets), Statistical significance of deviation of a given point from an unconstrained linear regression model comparing to +Dox with –Dox datasets was defined as *P* values calculated by *t*-test and adjusted by Benjamini-Hochberg FDR correction.

### MS-based metabolomics

Metabolite extraction for liquid-chromatography-mass spectroscopy (LC-MS) was performed on MEFs and human primary fibroblasts cultured in galactose medium for 48 h or 7 d, respectively. Cells were washed once with cold PBS (CST) and lysed at a concentration of 2 × 10^6^ cells/mL in a metabolite extraction buffer (50% methanol (Fisher Scientific), 30% acetonitrile (Sigma), 20% dH_2_O). Samples were vortexed for 45 s, centrifuged at 16,100 g and supernatants were subjected to LC-MS as follows, using a three-point calibration curve with universally labeled carbon-13/nitrogen-15 amino acids for quantification. Prepared samples were analysed on an LC-MS platform consisting of an Accela 600 LC system and an Exactive mass spectrometer (Thermo Scientific). A Sequant ZIC-pHILIC column (4.6 mm x 150 mm, 5 µm) (Merck) was used to separate the metabolites with the mobile phase mixed by A = 20 mM ammonium carbonate in water and B=acetonitrile. A gradient program starting at 20% of A and linearly increasing to 80% at 30 min was used followed by washing (92% of A for 5 mins) and re-equilibration (20% of A for 10 min) steps. The total run time of the method was 45 min. The LC stream was desolvated and ionised in the HESI probe. The Exactive mass spectrometer was operated in full scan mode over a mass range of 70–1,200 m/z at a resolution of 50,000 with polarity switching. The LC-MS raw data was converted into mzML files by using ProteoWizard and imported to MZMine 2.10 for peak extraction and sample alignment. A house-made database integrating KEGG, HMDB, and LIPID MAPS was used for the assignment of LCMS signals by searching the accurate mass and the metabolites used in the manuscript were confirmed by running their commercial standards. Finally, peak areas were used for comparative quantification. Output from MS-based metabolomics was subjected to statistical analysis by MetaboAnalyst 5.0. The variables were normalised by auto-scaling (mean-centered and divided by SD of each variable) by the MetaboAnalyst platform and then subjected to principal component analysis (PCA), heatmap and hierarchical clustering analysis with Euclidean distance, and complete-linkage method. Statistical significance was determined using the Student’s *t-*test corrected with the false discovery rate (FDR) method of Benjamini and Hochberg.

### Seahorse assay

MEFs seeded into Seahorse XF24 V7 assay plates (0.8 × 10^4^ cells/well) (Agilent Technologies, 100777-004) were cultured in glucose medium for 20 h. The media was switched to unbuffered glucose medium (DMEM (Sigma-Aldrich, D5030), 3% FBS, 5 mM glucose, 1 mM sodium pyruvate, 2 mM L-glutamine, 1 mM HEPES, pH 7.4) 1 h before the assay, and the plate was incubated at 37 °C without CO_2_. Oxygen consumption rates (OCR) and extracellular acidification rates (ECAR) were determined using Seahorse XF24 analyzer (Agilent Technologies) in the presence of different respiratory and glycolysis inhibitors which were sequentially added as follows: 1.5 µM oligomycin, 3 µM Carbonyl cyanide-4-(trifluoromethoxy)phenylhydrazone (FCCP), and a mixture of 0.5 µM Rotenone, 2.5 µM Antimycin A, and 50 mM 2-deoxyglucose. Cellular energetics for ATP production rates by mitochondrial oxidative phosphorylation and glycolysis were calculated by using the OCR and ECAR based on the methods described [62, 63], taking into account the acidification rates due to mitochondrial CO_2_ production, and were adjusted to protein concentration determined by the DC protein assay (BioRad). To measure CI- and CII-linked respiration, cells were permeabilised using Seahorse XF Plasma Membrane Permeabilizer (Agilent Technologies), and OCR was measured in the assay buffer (115 mM KCl, 10 mM KH2PO4, 2 mM MgCl2, 3 mM HEPES, 1 mM EGTA and 0.2% fatty acid-free BSA, pH 7.2, at 37 °C) with CI substrates (10 mM pyruvate and 1 mM malate) or CII substrate (4 mM succinate and 0.5 µM rotenone). During analysis, the following compounds were added to test mitochondrial activity and cellular bioenergetics flux: 4 mM ADP, 0.5 µM oligomycin, 2.5 µM FCCP, and 2.5 µM antimycin A.

To measure OCR in galactose culture, MEFs were seeded into Seahorse xFe96/XF Pro assay plates (0.8 × 10^4^ cells/well) (Agilent Technologies, 103794-100). Following 20 h culture in galactose medium, the media was switched to unbuffered galactose medium (DMEM (Sigma, D5030) 3% FBS, 10 mM Galactose, 1 mM Pyruvate, 2 mM L-Glutamine, 1 mM HEPES, pH 7.4), and the plate was incubated at 37 °C without CO_2_ for 1 h. OCR was determined using the Seahorse XFe96 analyzer (Agilent Technologies). During analysis, the following respiratory inhibitors were added sequentially: 2 µM oligomycin, 2 µM FCCP, and a mixture of 0.5 µM rotenone and 2.5 µM antimycin A. OCR data was normalized to cell content/well by staining cells with 0.2% crystal violet. Stained cells were lysed with 1% SDS, and the absorbance of the resulting solution was measured at 595 nm using a microplate reader (FLUOstar Omega, BMG Labtech).

### Electron Microscopy

MEFs seeded in a 6-well plate (0.3 × 10^6^ cells/2 mL/well) were cultured in galactose medium for 20 h. Cells were trypsinised, washed, collected and fixed overnight in 2% glutaraldehyde in 0.1 M cacodylate buffer. After rinsing in buffer, the cells were post-fixed in 1% osmium tetroxide + 1.5% potassium ferricyanide, rinsed in deionized water then dehydrated through a graded series of acetone. Cells were infiltrated with epoxy resin (TAAB medium) and polymerized at 60 °C for 36 h. Ultrathin sections (70 nm) were picked up on copper grids and stained with uranyl acetate and lead citrate before being viewed on a 100 kV CM100 TEM (FEI). Images of 10 cells per cell line were collected and quantified. Mitochondrial morphology in a slice was scored as either ‘normal’ or ‘abnormal’ (swollen or disputed cristae structure) and expressed as a ratio of mitochondria with ‘abnormal’ morphology per cell.

### NAD^+^ and NADH measurements

Measurements of NAD^+^ and NADH in mammalian whole-cell lysates, and in brain and liver mouse tissues were performed as described in a published protocol [8, 64]. NAD^+^ or NADH was extracted from 4 × 10^6^ cells (NAD^+^) or 8 × 10^6^ cells (NADH) cells by probe sonication with an acidic solution (10% trichloroacetic acid (TCA) (Sigma-Aldrich) or basic solution (0.5 M sodium hydroxide (Sigma-Aldrich), 5 mM EDTA (Sigma-Aldrich)) respectively. NADH samples were heated at 60 °C for 30 min. Samples were centrifuged at 16,100 g for 3 min at 4 °C. Supernatants were collected and 10% volume of 1 M Tris (Sigma-Aldrich) was added to adjust pH, followed by NAD^+^ and NADH measurements. A small amount of supernatant (NADH) or the pellet (NAD^+^) resolved in 0.2 M sodium hydroxide were used to measure protein concentrations. NAD^+^ and NADH levels were determined by the fluorescence intensity of resorufin produced by an enzymatic cycling reaction using resazurin, riboflavin 5′-monophosphate, alcohol dehydrogenase, and diaphorase (all from Sigma-Aldrich). Fluorescence intensity was monitored every minute for a total 60 min using a microplate reader (FLUOstar Omega, BMG Labtech). NAD^+^ and NADH levels were determined by a β-NAD (Sigma-Aldrich) standard curve and adjusted to protein concentration determined by the DC protein assay (BioRad).

### Image acquisition

Fluorescence images were obtained using an inverted DMi8 microscope (Leica) with a Plan-Apochromat 63x/1.40 oil immersion lens, equipped with an ORCA-Flash4v2.0 camera (Hamamatsu) (MitoSOX and mt-mKeima imaging), an Axio observer Z1 microscope (Zeiss), with a Plan-Apochromat 20x/0.8 M27 air immersion objective, equipped with an Axiocam 503 camera (LC3B staining in human primary fibroblasts), EVOS FL Cell Imaging System (Thermo Fisher Scientific) with AMG 10x Plan FL and AMG 40x Plan FL lens (iPSC experiments), or an LSM700 microscope (Zeiss) with a C-Apochromat 40x/1.20 water immersion lens (mitochondrial ΔΨm measurements in MEFs). Deconvolved images were generated using Huygens Essential software (version 20.10, Scientific Volume Imaging). Images were analyzed in Fiji/ImageJ (version 1.53c; NIH), and quantification was performed on at least 50 cells per condition.

### ROS measurement

Cells seeded in a 35 mm glass bottom dish (MatTek) were co-stained with 2.5 μM mitoSOX (Invitrogen, M36008) and 100 nM MitoTracker Green (Invitrogen, M7514) for 10 min and washed three times with galactose medium. Fluorescence images were obtained described as above. Fluorescence intensity was analysed as outlining single cells as regions of interest and calculation of the raw integrated density value per cell.

For the flow cytometry-based ROS analysis, human primary fibroblasts were cultured in galactose medium for 7 d, seeded in 6-well plate (0.15 × 10^6^ cells/well) and cultured in galactose medium for 48 h. Cells were stained with 10 µM CM-H_2_DCFDA (Invitrogen, C6827) for 30 min in the dark at 37 °C. Cells were then trypsinized and re-suspended in FACS sorting medium (3% FBS (BioSera) in PBS). Flow cytometry data (10,000 counts) was acquired on BD FACSCanto™ benchtop analyser (BD Biosciences), and analysed using the FlowJo software.

### Mitochondrial **ΔΨm** measurements

MEFs were grown in a 96-well glass bottom plate (Greiner Bio-One) (0.5 × 10^4^ /100 μL/well, 24 h). Following culture in galactose medium for 68 h, cells were co-stained with 16.7 nM tetramethylrhodamine methyl ester (TMRM; Invitrogen, T668) and 100 nM Mitotracker Green (MTG) for 30 min in conditioned galactose medium (68 h culture on *Npc1*^*-/-*^ MEFs, collected and filtered through a 0.22 µm pore-size filter). Live cell imaging was performed in a maintained atmosphere of 37 °C and 5% CO_2_. TMRM and MTG raw integrated density values per cell were quantified by outlining single cells as regions of interest. Mitochondrial ΔΨ was expressed as a ratio of TMRM to MTG. Quantification was performed on at least 30 cells per condition.

Measurements of ΔΨm in iPSC-derived cortical neurons were performed with TMRE (Invitrogen, T669) staining [65]. Cells were loaded with Microscopy Medium comprising 120 mM NaCl, 3.5 mM KCl, 0.4 mM KH2PO4, 5 mM NaHCO3, 1.2 mM NaSO4, 20 mM HEPES and 15 mM glucose in dH2O adjusted to pH 7.4 and supplemented with 1 mM CaCl2 (all from Sigma-Aldrich), and incubated with 500 nM TMRE for 1 h at 37 °C. The fluorescence signals of TMRE was acquired using EnSpire Multimode microplate reader (PerkinElmer) for a period of 5 min to get basal fluorescence, and again for TMRE for another 5 min after the addition of 10 μM FCCP (fluorocarbonyl cyanide phenylhydrazone). The baseline fluorescence was calculated as the mean of the last 5 fluorescence readings before the addition of FCCP, and the delta (Δ) fluorescence was calculated by subtracting the basal fluorescence from the average of first 5 fluorescence readings after FCCP treatment. Data were obtained as relative fluorescence units, normalised to protein concentration via Bio-Rad Protein Assay (Bio-Rad), and expressed as a percentage of the control condition.

### Immunoblotting

Immunoblotting was performed as described previously [66]. In brief, cells were lysed in RIPA buffer (Sigma-Aldrich) supplemented with 1× Halt™ protease and phosphatase inhibitor cocktail (Thermo Fisher Scientific). Protein concentration was measured using DC Protein Assay (Bio-Rad), and samples were prepared by boiling in Laemmli sample buffer (Bio-Rad) in the presence of 2.5% β-mercaptoethanol. Equal amounts of protein (20–40 μg) were subjected to SDS‐PAGE and transferred to PVDF membranes. Membranes were first blocked in 5% milk (Merck Millipore) in PBS with 1xTween® 20 (Sigma-Aldrich) for 1 h at room temperature and incubated with primary antibodies overnight at 4 °C. Secondary antibodies conjugated to horseradish peroxidase (HRP) for rabbit (Sigma-Aldrich, A0545) or mouse (Sigma‐Aldrich, A2554) were used at 1:5,000 dilution for 1 h at room temperature. Chemiluminescence detection was achieved using Clarity Western ECL Substrate (Bio-Rad) and a LAS4000 CCD camera system (Fujifilm) or an iBright CL1500 imaging system (Invitrogen). The following primary antibodies were used:

β-actin (St John’s Laboratory, STJ96930, 1:5000), Cleaved caspase-3^Asp175^ (CST, 9661, 1:250), HaloTag (Promega, G9211, 1:2000), LC3B (CST, 3868, 1:1000), NPC1 (abcam, ab134113, 1:1000), phospho-p70S6K^Thr389^ (CST, 9234, 1:1000), p70S6K (CST, 9202, 1:1000), phospho-ULK1^Ser757^ (CST, 6888. 1:1000). Densitometry analyses of immunoblots were done using Fiji/ImageJ (version 1.53c; NIH).

### Immunofluorescence

Human primary fibroblasts seeded onto 13 mm coverslips were cultured for 48 h. Cells were fixed and permeabilised in −20 °C 100% methanol for 5 min and blocked in 5% normal goat serum in PBS for 1 h and incubated with LC3B antibody (CST, 3868S, 1:1000) overnight at 4 °C. Cells were washed and incubated with Alexa Fluor 488 goat anti-rabbit (H + L) antibody (1:1000; Thermo Fisher Scientific; A-11008) for 1 h at room temperature. Coverslips were mounted on slides with Prolong Gold antifade reagent with DAPI (Invitrogen, P36931).

iPSC-derived cortical neurons were washed in PBS, fixed with 4% formaldehyde at room temperature for 15 min, permeabilised with 0.5% Triton X-100 for 10 min (except LC3B staining) or with pre-chilled methanol for 5 min (for LC3B antibody), and incubated with Blocking Buffer (5% goat or donkey serum (Sigma-Aldrich) in PBS with or without (LC3B staining) 0.05% Tween 20 for 1 h at room temperature. Cells were then incubated overnight with primary antibodies at 4 °C, followed by incubation with Alexa Fluor conjugated secondary antibodies for rabbit (Thermo Fisher Scientific, A21206) or mouse (Thermo Fisher Scientific, A21203) for 1 h at room temperature. The coverslips were mounted on glass slides with ProLong Gold antifade reagent with DAPI (Invitrogen). The following primary antibodies were used: rabbit α-LC3B (Novus Biologicals, NB100-2220, 1:200), mouse α-p62 (BD Biosciences, 610832, 1:200), rabbit α-MAP2 (CST, 8707, 1:200) mouse α-TUJ1 (CST, 4466, 1:200).

### Cell death assays

Adherent and floating MEFs were collected and processed by protein extraction and immunoblot analysis at 24 h (*Atg5*^*+/+*^ and *Atg5*^*-/-*^ MEFs) and 72 h (*Npc1*^+/+^ and *Npc1*^*−/−*^ MEFs) after media switch. Representative phase-contrast images were obtained on an inverted DM-IL Leica microscope equipped with an Invenio 3SII digital camera (3.0 Mpix Colour CMOS; Indigo Scientific).

Cytotoxicity was measured using Cytotox-Glo Cytotoxicity Assay (Promega, G9291) according to manufacturer instruction. Cells in a 96-well white plate (Greiner) cultured for the indicated times after media switch were incubated with Cytotox-Glo Assay Reagent for 15 min at room temperature in the dark, then luminescence was measured using a GloMax plate-reader (Promega) or EnSpire Multimode plate reader (PerkinElmer) and the readings obtained were attributed to the basal cytotoxicity per well (first reading). To estimate cell population per well, cells were further incubated with Lysis Reagent for 30 min at room temperature in the dark, after which luminescence was measured again (second reading). Cytotoxicity data were normalised by dividing the first reading (basal cytotoxicity per well) to the second reading (indicative of cell population per well) and expressed as a percentage.

TUNEL staining for apoptotic neuronal cells were performed using Click-iT Plus TUNEL Assay kit (Invitrogen, C10617), according to the manufacturer’s protocol. For detection of TUNEL^+^ apoptotic nuclei specifically in neurons, cells were subjected to immunofluorescence by blocking with 3% BSA (in PBS) followed by incubation with anti-TUJ1 antibody (BioLegend, 801201) overnight at 4 °C, and thereafter incubated with Alexa Fluor 594 secondary antibody for 1 h at room temperature. Coverslips were mounted on glass slides with ProLong Gold antifade reagent with DAPI (Invitrogen). The quantification of TUNEL^+^ apoptotic nuclei in TUJ1^+^ neuronal cells was performed via fluorescence microscopy, as previously described [20]. The percentage of TUNEL^+^ nuclei was calculated from the total number of TUJ1^+^ cells analysed.

### Assessment of autophagy

LC3B turnover assay was performed to monitor autophagy flux in MEFs and human primary fibroblasts[34]. Cells were cultured in galactose medium in the presence or absence of Baf_A1_ (to block lysosomal degradation) for 24 h, followed by immunoblotting. LC3B-II flux was expressed by subtracting the signals of LC3B in Baf_A1_-untreated conditions from those of LC3B in Baf_A1_-treated conditions. Additionally, LC3B-positive autophagosomes in human primary fibroblasts were visualised by immunofluorescence analysis. The number of LC3B puncta per cell was quantified.

Halo processing assay was conducted to assess autophagy-inducing activities of small molecules [35]. *Npc1*^*-/-*^ MEFs expressing Halo-GFP-LC3B were treated with compounds and 20 µM 7-Bromo-1-heptanol (HaloTag-blocking agent) (Thermo Scientific Chemicals, H54762) [67], followed by immunoblot analysis using Halo antibody. Autophagic activity was quantified as a ratio of Halo monomer per Halo-GFP-LC3B.

In iPSC-derived cortical neurons, autophagic activity was assessed by the degradation of autophagy marker proteins, p62 and LC3B, by immunofluorescence. The number of LC3B or p62 puncta in TUJ1- or MAP2-positive cells was quantified.

### Mitophagy assay

MEFs expressing YFP-Parkin and human primary fibroblasts were transduced with mt-mKeima as above. Cells were seeded in a 35 mm glass bottom dish, and the live-cell mt-mKeima signal was obtained using a DMi8 microscope (Leica). Mitophagy events were determined as following steps using Fiji/ImageJ (version 1.53c; NIH) [68]. Images were masked by applying MaxEntropy threshold algorithm to the images obtained with 561 nm excitation to remove low red signal and background. Within the masks, signals of mt-mKeima were adjusted by applying Enhanced Contrast plugin with saturated = 0.1, normalise, equalise options. Then, images were generated by subtracting the signal at a 480 nm excitation (reporting neutral pH-environment) from the signal at a 561 nm excitation (reporting an acidic pH-environment). Resulting images were binarised with the MaxEntropy threshold algorithm to extract mitolysosomes. The number of puncta per cell in images was quantified by outlining single cells as regions of interest and counted using Analyze Particles plugin.

### Statistical analyses

All experiments were carried out in three or more biological replicates from independent cell culture or cell lines (human primary fibroblasts). Quantifications of data and statistical analysis of metabolomics are described under the corresponding method section. Sample size calculation was not performed. Graphical data denote the mean ± SEM (of n = 3 or more biological replicates or cell lines) and are depicted by column graph scatter dot plot, using Prism 8.4.3 software (GraphPad). Unless indicated otherwise, the *P* values was determined by Student’s *t-*test (two-tailed, unpaired) between two groups or by one-way ANOVA followed by multiple comparisons with the two-stage linear step-up procedure of Benjamini, Krieger, and Yekutieli (with an FDR value of 0.05) using Prism 8.4.3 software (GraphPad). *, *P* < 0.05; **, *P* < 0.01; ***, *P* < 0.001; ns (non-significant).

### Supplementary information

Supplementary information

Original western blots

## Data Availability

Original images of immunoblotting are provided as supplementary material. The mass spectrometry metabolomics source data can be accessed via the following link: ftp://massive.ucsd.edu/v07/MSV000094506/. All other datasets generated in this study are presented and analysed within this manuscript and are available from the corresponding authors upon request.
