# Supplementary material for: Targeting the autophagy-NAD axis protects against cell death in Niemann-Pick type C1 disease models
Source: Cell Death Dis. 2024 May 31;15(5):382. doi: 10.1038/s41419-024-06770-y (PMC11143325; doi:10.1038/s41419-024-06770-y)
Supplement: Supplementary file 1 — Supplementary information [file 41419_2024_6770_MOESM1_ESM.pdf]

Supplementary Figure and Legend

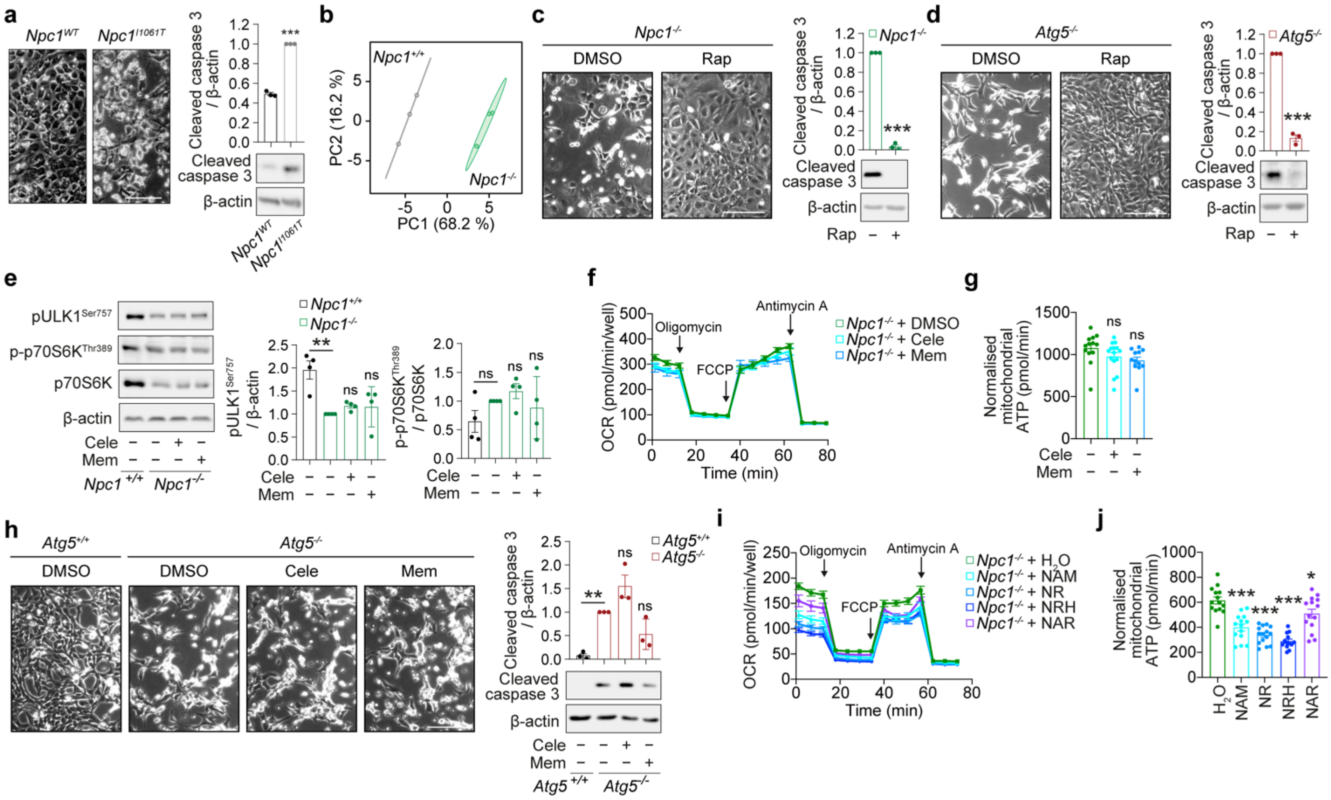

Figure S1. Cellular phenotypes caused by the loss of NPC1 or ATG5 function and in response to pharmacological interventions.

a, Phase-contrast images and immunoblot analysis for caspase-3 cleavage of primary MEFs isolated from NPC1 (*Npc1*^{I1061T}) and control (*Npc1*^{WT}) mice, cultured in galactose medium for 168 h. **b,** Principal component analysis (PCA) of metabolomics datasets in Fig. 1d, e. **c, d,** Phase-contrast images and immunoblot analyses for caspase-3 cleavage of *Npc1*$^{-/-}$ MEFs (c) or *Atg5*$^{-/-}$ MEFs (d) cultured in galactose medium supplemented with 100 nM rapamycin (Rap) for 72 h (c) or 24 h (d). **e,** Immunoblot analyses of mTOR signalling in *Npc1*$^{+/+}$ and *Npc1*$^{-/-}$ MEFs cultured in galactose medium supplemented with 10 μM celecoxib (Cele) or 30 μM memantine (Mem) for 24 h. **f, g,** Oxygen consumption rate (OCR) (f) and mitochondrial ATP production rate (g) of *Npc1*$^{-/-}$ MEFs cultured in galactose medium supplemented with Cele or Mem for 24 h. **h,** Phase-contrast images and immunoblot analysis for caspase-3 cleavage of *Atg5*$^{+/+}$ and *Atg5*$^{-/-}$ MEFs cultured in galactose medium

supplemented with 10 μM Cele or 30 μM Mem for 24 h. **i, j,** OCR (i) and mitochondrial ATP production rate (j) of *Npc1*$^{-/-}$ MEFs cultured in galactose medium supplemented with 5 mM nicotinamide (NAM), 2 mM nicotinamide riboside (NR), 300 μM reduced nicotinamide riboside (NRH) and 50 μM nicotinic acid riboside (NAR) for 24 h.

Data are mean ± SEM of n = 3 or 4 biological replicates (**a, b, c, d, e, h**) or n = 12-15 technical replicates (**f, g, i, j**) as indicated. *P* values were calculated by unpaired two-tailed Student's *t*-test (**a**, **c**, **d**) and by one-way ANOVA followed by multiple comparisons with the two-stage linear step-up procedure of Benjamini, Krieger and Yakutieli (**e, g, h, j**). *P<0.05; **P<0.01; ***P<0.001; ns (non-significant) with respect to WT (**a**), untreated *Npc1*$^{-/-}$ (**c**, **e, g, j**) or *Atg5*$^{-/-}$ (**d**, **h**) MEFs. Scale bar: 200 μm (**a, c, d, h**).
