# Supplementary figures and images for: Targeting the autophagy-NAD axis protects against cell death in Niemann-Pick type C1 disease models
Source: Cell Death Dis. 2024 May 31;15(5):382. doi: 10.1038/s41419-024-06770-y (PMC11143325; doi:10.1038/s41419-024-06770-y)

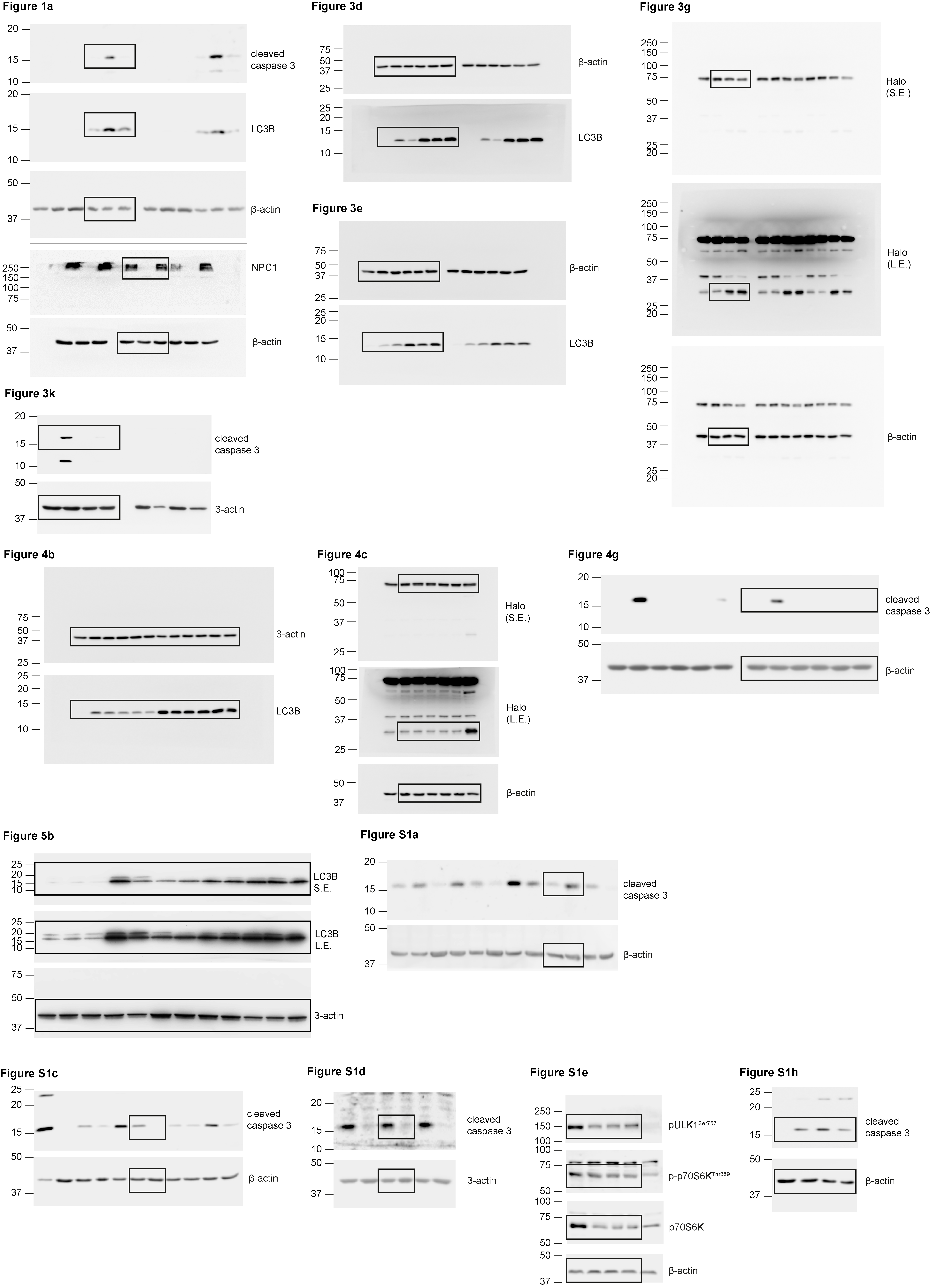

Supplement: Supplementary file 2 — Original western blots [file 41419_2024_6770_MOESM2_ESM.png]
